# Analysis of viral diversity for vaccine target discovery

**DOI:** 10.1186/s12920-017-0301-2

**Published:** 2017-12-21

**Authors:** Asif M. Khan, Yongli Hu, Olivo Miotto, Natascha M. Thevasagayam, Rashmi Sukumaran, Hadia Syahirah Abd Raman, Vladimir Brusic, Tin Wee Tan, J. Thomas August

**Affiliations:** 1 Centre for Bioinformatics, School of Data Sciences, Perdana University, Jalan MAEPS Perdana, Serdang, Selangor Darul Ehsan 43400 Malaysia; 2 Department of Pharmacology and Molecular Sciences, The Johns Hopkins University School of Medicine, 725 North Wolfe Street, Baltimore, MD 21205 USA; 3 Department of Biochemistry, Yong Loo Lin School of Medicine, National University of Singapore, 8 Medical Drive, Singapore, 117597 Singapore; 4 Centre for Genomics and Global Health, University of Oxford, Oxford, UK; 5 Mahidol-Oxford Research Unit, Faculty of Tropical Medicine, Mahidol University, Rajthevee, Bangkok, Thailand; 6 Menzies Health Institute Queensland, Griffith University, Parklands Dr, Southport, 4215 QLD Australia

**Keywords:** Viral diversity, Bioinformatics, Vaccine design, Target discovery, Reverse vaccinology, Database, Tools

## Abstract

**Background:**

Viral vaccine target discovery requires understanding the diversity of both the virus and the human immune system. The readily available and rapidly growing pool of viral sequence data in the public domain enable the identification and characterization of immune targets relevant to adaptive immunity. A systematic bioinformatics approach is necessary to facilitate the analysis of such large datasets for selection of potential candidate vaccine targets.

**Results:**

This work describes a computational methodology to achieve this analysis, with data of dengue, West Nile, hepatitis A, HIV-1, and influenza A viruses as examples. Our methodology has been implemented as an analytical pipeline that brings significant advancement to the field of reverse vaccinology, enabling systematic screening of known sequence data in nature for identification of vaccine targets. This includes key steps (i) comprehensive and extensive collection of sequence data of viral proteomes (the virome), (ii) data cleaning, (iii) large-scale sequence alignments, (iv) peptide entropy analysis, (v) intra- and inter-species variation analysis of conserved sequences, including human homology analysis, and (vi) functional and immunological relevance analysis.

**Conclusion:**

These steps are combined into the pipeline ensuring that a more refined process, as compared to a simple evolutionary conservation analysis, will facilitate a better selection of vaccine targets and their prioritization for subsequent experimental validation.

**Electronic supplementary material:**

The online version of this article (10.1186/s12920-017-0301-2) contains supplementary material, which is available to authorized users.

## Background

Novel vaccine design strategies are required to overcome the deficiency in the immune response to pathogens that do not naturally result in lasting immunity [1], such as human immunodeficiency virus (HIV), influenza, dengue, and hepatitis C, among others. A vaccine that simply mimics the antigens of the natural pathogen has little chance of being successful, unless the immune deficiency is the result of some viral mechanism that can be overcome by an attenuated or inactivated pathogen. This is clearly not the case with the extensively studied HIV-1 where virus replication, while initially inhibited by the vaccine, is rapidly resumed by mutation and the selection of immune escape variants [2].

We have confronted this problem of viral diversity through in-depth quantitative analyses of the pathogen proteome, including the dynamics of protein mutations in the context of T-cell immunity, diversity of the immune relevant overlapping nonamer (residues 1–9, 2–10, 3–11, etc.; represent the binding core length in T-cell recognition) peptide sequences of the proteins, the intra- and inter-species similarities of the nonamer sequences (covering matches with human tissue proteins), and host immune responses to the nonamers. The approach takes advantage of the large quantities of pathogen sequence data produced by genomics, proteomics, and host-pathogen interaction studies that are made available in public repositories and the data are continuing to grow rapidly. Viral data accumulated over the years have resulted in large data sets, in the range of tens or even hundreds of thousands of reported protein sequences, such as of dengue, HIV-1 and influenza A viruses. In many cases, the application of such large data sets is critical for vaccine target discovery as they enable detailed analysis of target antigens and their roles in the immune response.

There are several sophisticated bioinformatics methods to deal with the combinatorial complexity of viral diversity and of the human immune system, including both the antibody and T-cell arms. Systematic bioinformatics approaches that combine multiple computational approaches are necessary for the analysis of large data sets and for selection of potential candidate vaccine targets that can be experimentally validated [3]. The goal is a systematic investigation of antigen sequences suitable as targets for vaccine formulation and for new insights into pathogen recognition by the immune system of the host [4, 5].

A common approach for study of antigenic diversity is evolutionary (phylogenetic) analysis [6]. Genetic diversity of viruses is related to antigenic diversity, however they are not the same. Some evolutionary differences at nucleotide level do not translate into difference at protein level due to synonymous mutations. Further, a single mutation may result in the loss of immune recognition of mutated target. Such effects are not captured by phylogenetic analysis. Several methodologies for vaccine formulation, designed to overcome viral sequence diversity have been reported. A common approach is the use of evolutionarily centralized or the consensus (most common) sequence of the viral population to minimize the variations between viral strains and the vaccine [7]. However, such sequences are often not good indicators of conservation. A recent modification of the centralized approach was to prepare sets of ‘mosaic’ proteins, derived from natural sequence fragments assembled into a small number of native-like proteins [8–10]. Although, concatenated mosaic sequences selected from conserved regions of proteins are better indicators of conservation, full-length mosaic vaccines, however, will require a large cocktail size for significant coverage of variability for pathogens of extreme diversity, such as HIV-1, where even the conserved regions are of some variability. Large cocktail size is not ideal for practical vaccine application.

Herein, we describe a computational methodology pipeline for characterization of potential T-cell epitopes of viral proteins, including the immune relevant diversity of the sequences [11]. We deployed this pipeline to map potential vaccine targets for dengue [12–14], West Nile [15], hepatitis A (unpublished work), HIV-1 [2], and influenza A viruses [16, 17]. The methodology includes the study of possible effects of antigenic variation and species sequence similarities to immune responses mediated by T cells, with consideration of the human leukocyte antigen (HLA, human MHC) polymorphism in the human population [18]. The results offer insights into antigenic diversity of viruses, in contrast to genetic diversity, and the impact of this diversity on effective strategies for vaccine design. The specific goals of this methodology include a strategy for rational selection of viral vaccine targets that cover antigenic diversity. A recent strategy for the study of antigenic diversity has been to focus on the identification of peptides of the virus species of interest that are both highly conserved across viral variants and immunologically relevant with the potential to bind multiple HLA alleles (promiscuous or HLA supertype-restricted peptides) [19, 20]. However, our study of viral diversity for vaccine target discovery has shown that there is often extensive sharing of immune relevant peptides of a given viral species of interest with multiple other viruses and organisms, such as sharing among *Flaviviruses* [12, 15]. These shared epitopes, while highly conserved in a given virus species of interest, are also present in many other viruses/organism, but typically not as a highly conserved sequence (i.e. low incidence or frequency among the reported sequences of the other species), resulting in the presence of inter-species altered peptide ligand (APL) sequences. These are peptides of the other species that are variant to the highly conserved, immune epitope of the species of interest by one or more amino acid differences [21, 22]. These APLs may pose potential risk of deleterious immune responses and immunopathology for subjects exposed to multiple viruses by infection or immunization. This may also be triggered by intra-species APLs of variable epitopes. An example is the case of secondary dengue infection [23–27] where pre-existing immunity is thought to result in immunopathology following infection by different serotype of the same viral species. The results of our analysis of multiple virus species suggest that it is important to use virus species-specific highly conserved, immune relevant sequences as vaccine targets for new generation vaccines. This approach is different to current approaches that are based on antigenic diversity analysis restricted to only the virus species of interest.

**Fig. 1:**
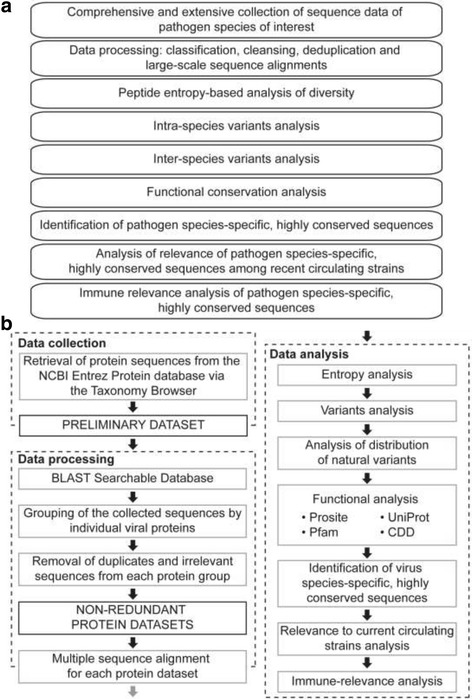
Overview of the methodology pipeline. **a** Summary of the main steps involved. **b** A workflow describing the main steps involved

The methodology pipeline (Fig. 1) brings significant advancement to the field of reverse vaccinology because it enables systematic screening of all reported sequence data for selection of virus species-specific vaccine targets. This represents a departure from the approaches where a small number of viral strains of the species of interest are studied with limited in silico analyses of conservation and variability to identify putative antigens as vaccine targets.

### Materials

The relevant bioinformatics tools, web servers, and tutorials described herein are collectively listed in Table 1 with the corresponding URLs.

**Table 1:** Bioinformatics tools, web servers and tutorials relevant as part of the vaccine target discovery pipeline described herein

Tool	URL (http or ftp)
AVANA	http://sourceforge.net/projects/avana/
BioEdit	http://www.mbio.ncsu.edu/bioedit/bioedit.html
BLAST Web server	http://blast.ncbi.nlm.nih.gov/
BLAST+	http://tinyurl.com/259v9pq
BLAST+ Databases	ftp://ftp.ncbi.nlm.nih.gov/blast/db/
BLAST+ Manual	http://tinyurl.com/njr9xk6
BLAST2GO	https://www.blast2go.com/
CDD	http://www.ncbi.nlm.nih.gov/Structure/cdd/cdd.shtml
ClustalX	ftp://ftp.ebi.ac.uk/pub/software/clustalw2/
HCV Immunology Database	https://hcv.lanl.gov/content/immuno/immuno-main.html
HIV Molecular Immunology Database	https://www.hiv.lanl.gov/content/immunology/
HIV Sequence Database	https://www.hiv.lanl.gov/
Immune Epitope Database	http://www.iedb.org/
Influenza Database	http://research4.dfci.harvard.edu/cvc/flukb/
Jalview	http://www.jalview.org
MHCBN	http://crdd.osdd.net/raghava/mhcbn/
MULTIPRED2	http://cvc.dfci.harvard.edu/multipred2/
Muscle	http://www.drive5.com/muscle/
NCBI Entrez Databases	http://www.ncbi.nlm.nih.gov
NCBI Entrez Protein Database	http://www.ncbi.nlm.nih.gov/protein
NCBI Entrez Taxonomy Database	http://www.ncbi.nlm.nih.gov/taxonomy
NetCTL	http://www.cbs.dtu.dk/services/NetCTL
PEPVAC	http://imed.med.ucm.es/PEPVAC/
Pfam	http://pfam.xfam.org/
PROMALS3D	http://prodata.swmed.edu/promals3d/promals3d.php
Prosite	http://www.expasy.org/prosite
R	http://www.r-project.org
SYFPEITHI	http://www.syfpeithi.de
Tutorial 1: create BLAST searchable database	http://tinyurl.com/4bl2qlv
Tutorial 2: remove duplicate sequences	http://tinyurl.com/yadxuqe
Tutorial 3: generate tabbed alignment file (in.taln format) for input to AVANA	http://tinyurl.com/6cjb4gg
Tutorial 4: Notes on performing BLAST search with short queries	http://tinyurl.com/4uw5nd9
UniProt	http://www.uniprot.org

## Methods and Results

### Data collection

The study of viral diversity involves the analysis of sequences and their past evolutionary history. It is, therefore, important, to collect all available sequences of the virus of interest from public databases to enable a comprehensive and detailed analysis of viral diversity across a broad spectrum of geographical and temporal distributions of the virus. The NCBI Entrez databases are a typical source for retrieval of the sequences due to their comprehensive collection, but the primary databases are generally prone to errors and incomplete annotations. The reported sequences of the virus species (protein or nucleotide) can be downloaded by use of a taxonomy ID or species name search via the NCBI taxonomy browser [28]. Searching through the taxonomy browser is superior to keyword search in the specific databases (for example NCBI Entrez Protein Database) as it takes away the need to search for all the synonyms of the virus species for a comprehensive collection, with minimum chances of false positive hits.

In some instances, specialized databases are available for the virus species of interest, for example HIV Sequence Databases. In such cases, the specialized database needs to be evaluated whether it is comprehensive, complete, up-to-date, and consists of high quality annotations. If these databases are not up-to-date, they should be complemented with entries from the NCBI databases. The specialized databases typically include additional functional or structural annotations. Suggested steps are as follows:

1. Search for the virus species name (e.g. “dengue virus”) or the taxonomy ID (e.g. “12637” for dengue virus) at the NCBI Entrez Taxonomy Database, and click on the organism name to access all types of available data for the species, such as protein, nucleotide, structure, genome sequences, among others (Fig. 2).

**Fig. 2:**
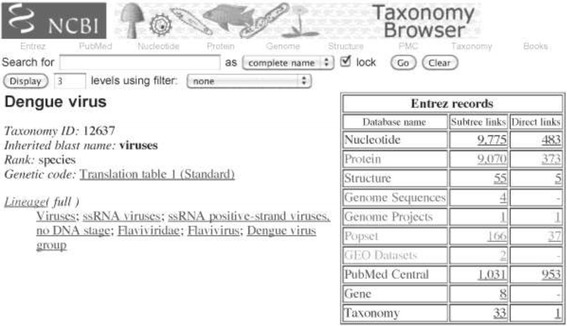
An example of a search result at the NCBI Entrez Taxonomy Database for data collection
2. We will focus on the protein sequences, which are accessible through the Protein hyperlink under the “Subtree links” column. These can be downloaded through the “Send to” dropdown menu, by selecting the “File” option under “Choose Destination”, with “Fasta” as the “Format”, followed by selecting the “Create File” button. Similarly, the full-records can be downloaded using the “GenPept (full)” as the format.

### Data processing

The collected sequences are classified according to the proteins encoded by the genome of the virus species of interest for ease in data processing and downstream analysis. The proteins of viruses with multiple distinct groups, such as HIV-1 clades or dengue serotypes or influenza A subtypes, are further sub-classified according to the individual groups, and a pan-group is also created for each of the proteins that comprises sequences of the different groups. The classification of the sequences by proteins is particularly important for viruses, such as *Flaviviruses*, where the RNA genome is translated into a single polyprotein, which is then cleaved by proteases to yield the individual proteins. The NCBI records for these viruses are generally of three types, namely (i) genome polyprotein records that provide the complete proteome as a single sequence, (ii) partial polyprotein records that provide a number of the encoded proteins as a single sequence, and (iii) individual proteins records that provide the sequence of only one protein. Viruses that do not translate into a single polyprotein are typically stored by NCBI as the category (iii) protein records. However, it should be noted that protein sequences of type (iii) records are often fragments that have not been completely sequenced. These partial sequences (at least nine amino acids long for immune relevance; see section “Entropy analysis”) can still be considered for viral diversity analysis as they provide some historical information. Although including these sequences provides more variant data, they can also be the cause for various sequence misalignments that will require manual inspection and correction.

Data compiled from the public databases are prone to errors and discrepancies [6, 29, 30], which may affect the classification of sequences into the different proteins and/or the subsequent analyses. Therefore, manual curation needs to be carried out, to ascertain reliability, and to correct errors if identified, including providing feedback to the source database to prevent propagation of such errors in the research work of others.

1. A BLAST [31] searchable database of the collected sequences is constructed to facilitate the classification by protein and virus group (clades/serotypes/subtypes). A sample full-length sequence for the proteins of each group (protein/group) is identified from the collected data and used as a query for the search. One sample sequence should be sufficient to capture all the variants within each group. Since public databases contain errors, it is critical to ascertain the accuracy of the sample sequences selected by cross-referencing with the literature. The parameters set for the BLAST search include: i) low-complexity filter off, ii) E-value threshold of 1, which may be increased to 10 or 100 if short partial protein sequence hits are expected from the searchable database (large E-value is necessary to detect matching short partial sequences), iii) number of descriptions and alignments set to a size larger than the total data in the searchable database, and iv) default setting for all other parameters, including the BLOSUM62 matrix which is usually sufficient to detect similar sequences of a protein. Local BLAST client program (BLAST+) can be downloaded from the URL provided in the “Materials” section. Construction of searchable databases is described in Tutorial 1 and BLAST+ user manual. The BLAST search result hits for each query sequence are manually inspected for reliability (analyzing the criteria and general guide: E-value (typically less than 0.05), alignment score (>50), % identity (>30%), % positive (the larger the better, >50%; will be at least equal to % identity), % query coverage (>50% would be good; but smaller maybe acceptable), and hit record annotations), with false positive hits (i.e. hits that do not meet the reliability criteria) rejected and the remaining sequences (all the hits sans the rejected ones) selected as the dataset for the protein/group.
2. The selected dataset of each protein/group is inspected again for errors and discrepancies and to filter out irrelevant sequences (sequences that meet the “good” BLAST hit criteria but may not be desired for certain reasons, such as if they do not meet a desired metadata criterion, for example year of isolation or certain lab strains). In this process, the following metadata should be extracted from the records and/or literature, and tabulated to facilitate downstream analyses: viral strain name, country isolated, year isolated, and the host species.
3. Bias may result from the collection of full-length or partial subsequences that are redundant [32], corresponding to identical or highly similar strains sequenced by various surveillance programs of different countries. Although duplicate sequences may represent the incidence of virus strains from nature, it is best to repeat the studies with non-redundant sequences in order to assess the potential bias effect (Tutorial 2 describes how to remove duplicates). Highly similar sequences should not be removed because an arbitrary rejection of such sequences may introduce additional bias. All the subsequent steps described below are applicable to both the redundant and non-redundant datasets of each protein/group of a virus species of interest.
4. All the individual protein/group and protein/pan-group datasets are then aligned. The MUSCLE program [33] is suitable for quick and reliable alignment; a local client, with usage documentations, can be downloaded from the URL; other tools may also be considered. The default alignment parameters are used because the resulting alignment has to be visually inspected and manually corrected for misalignments, using either the BioEdit Sequence Alignment Editor [34, 35] or Jalview [36]. The reliability of the alignment is assessed by ensuring correct positioning of alignment anchors, such as known conserved domains, motifs and/or cysteine residues forming disulphide bridges (known to remain conserved), as well as referencing existing published alignments. Alignment may be difficult for: i) viruses with highly diverse proteins, such as the haemagglutinin (HA) and neuraminidase (NA) proteins of influenza A virus [16], and envelope (Env) protein of HIV-1 [2] and dengue [12]; ii) viruses with large number of variant sequences, such as Env and Nef of HIV-1 or HA and NA of influenza A; and iii) viruses with large number of partial sequences, such as Env of dengue. Misalignments due to partial sequences can usually be corrected by manual editing when a reliable reference alignment is available. A divide and conquer approach is employed in the case of proteins that are highly diverse or are represented by large number of sequences, including application of combinations of different alignment programs. For example, proteins with large dataset are first split into smaller and more manageable regions (about 200–500 sequences per region). These smaller region subsets are then aligned using MUSCLE, PROMALS3D [37] or CLUSTAL W/X [38] for the most reliable alignment, which is then refined with RASCAL [39] before merging the subsets into a full protein multiple sequence alignment by use of conserved domain/motifs as anchors. The final protein alignments are manually inspected and corrected for misalignments. All individual alignment positions that are predominantly made up of gaps (95% or more) are removed as it is difficult to ascertain their reliability.

Some of the above steps require a number of unspecified manual error corrections. Often the heuristic nature of the methods used, such as multiple sequence alignment, require such a treatment. The risk of this semi-formal methodology, with the lack of formal demonstration of validity, quality checks, and sensitivity analysis, among others, is generally limited in returning reproducible results. For example, in the case of manual inspection of BLAST hits, several criteria are used to assess their reliability, increasing the chance of the same decision being taken at different occasions of inspection or when a different parameter value maybe used. Similarly, correcting misalignments (largely a result of partial sequences), although may seem more subjective, is guided by the principle of finding parts of the misaligned sequence among the other homologous sequences of the multiple sequence alignment. Conserved parts of the whole alignment often act as good anchors in correcting the misalignments. Where the misalignments are a result of high variability, such regions are usually not useful for vaccine design.

### Entropy analysis

T-cell epitopes are short peptides, typically with a core binding length of nine amino acids (nonamers; *9*-mers), where one or multiple amino acid changes in the composition can produce combinatorial diversity even when neighboring sites are highly conserved; a single amino acid change in a nonamer epitope can affect eight other overlapping nonamers [2]. Widely used approaches for studying viral diversity rely on the analysis of individual amino acid positions of an alignment. A more robust method is based on Shannon’s entropy [40, 41] to measure the degree of conservation and variability of peptides of desired length, and infer their evolutionary stability. For immunological applications, entropy measure for viral sequences is based on overlapping nonamer peptides (i.e. 1–9, 2–10, 3–11, *etc*) [42], and the method is described in [12]. Briefly, the computation of entropy at a given nonamer position involves the interplay of two factors: the number and incidence of distinct nonamer peptides. Nonamer entropy *H(x)* for a given site *x* in the alignment is calculated using the formula:

$$ H(x)=-\sum \limits_{i=1}^{n(x)}{p}_{i,x}{\log}_2\left({p}_{i,x}\right) $$

where *p*_*i,x*_ is the probability of the incidence of a nonamer *i* with its center position at *x* (for overlapping nonamer position 1–9, the center position *x* would be 5), while *n(x)* is the total number of unique nonamers at *x*. Only nonamers that contain a valid amino acid (excluding B, asparagine or aspartic acid; J, leucine or isoleucine; Z, glutamine or glutamic acid; and X, unknown amino acid) at position *x* are considered for the entropy computation, and those containing only gaps, observed due to presence of partial sequences in the alignment, are ignored. If the incidence of nonamers containing only gaps at position *x* is more than 50% of the sequences in the alignment, the position is discarded because of reduced statistical support, resulting in artificially low entropy value. Since entropy calculation is centered to a given nonamer position, no entropy values will be plotted for the first and the last four amino acids of an alignment.

**Fig. 3:**
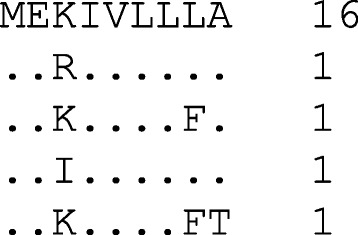
A sample nonamer position *x* with five distinct nonamer sequences and their incidences

The entropy calculation for a sample nonamer position *x* (Fig. 3) using the above formula is as follows:

$$ H(x)=-\left(4\ \mathrm{x}\ \frac{1}{20}{\log}_2\frac{1}{20}\ \right)+\left(\frac{16}{20}{\log}_2\frac{16}{20}\right)=0.606843 $$

An example of a high entropy nonamer position is one with a large number of distinct peptides, where the majority exhibit low incidence. Sites with high entropy imply high diversity, where the maximum theoretical value possible is 39 (log_2_ 20^9^), however, naturally occurring viral sequences show much lower entropy values than this because sequences of the same species share some level of conservation. Analysis of highly diverse HIV-1 clade B protein sequences revealed a peak entropy value of ~10 [2]. In contrast, the minimum possible entropy value is zero, where only a single distinct peptide with 100% incidence is observed at the nonamer site.

The number of sequences in an alignment (small versus large size) can affect the entropy calculations, and thus nonamer entropy values are corrected for size bias via a statistical sub-sampling method as described in [12]. Briefly, given the relationship that alignment size bias is proportional to 1/N where N is the alignment size [43], correction for size bias for a nonamer position *x* can be made through repeated random sampling of the sequences at the position, to create smaller alignments of varying size whose entropies are measured. A plot of these entropy values against 1/N allows extrapolation of entropy to N → ∞ by use of a linear regression and its coefficient of determination (r^2^) is used to measure the goodness-of-fit. The computation of entropy, including the correction for size bias has been implemented in our freely available Antigenic Variability Analyser tool (AVANA) [44].

**Fig. 4:**
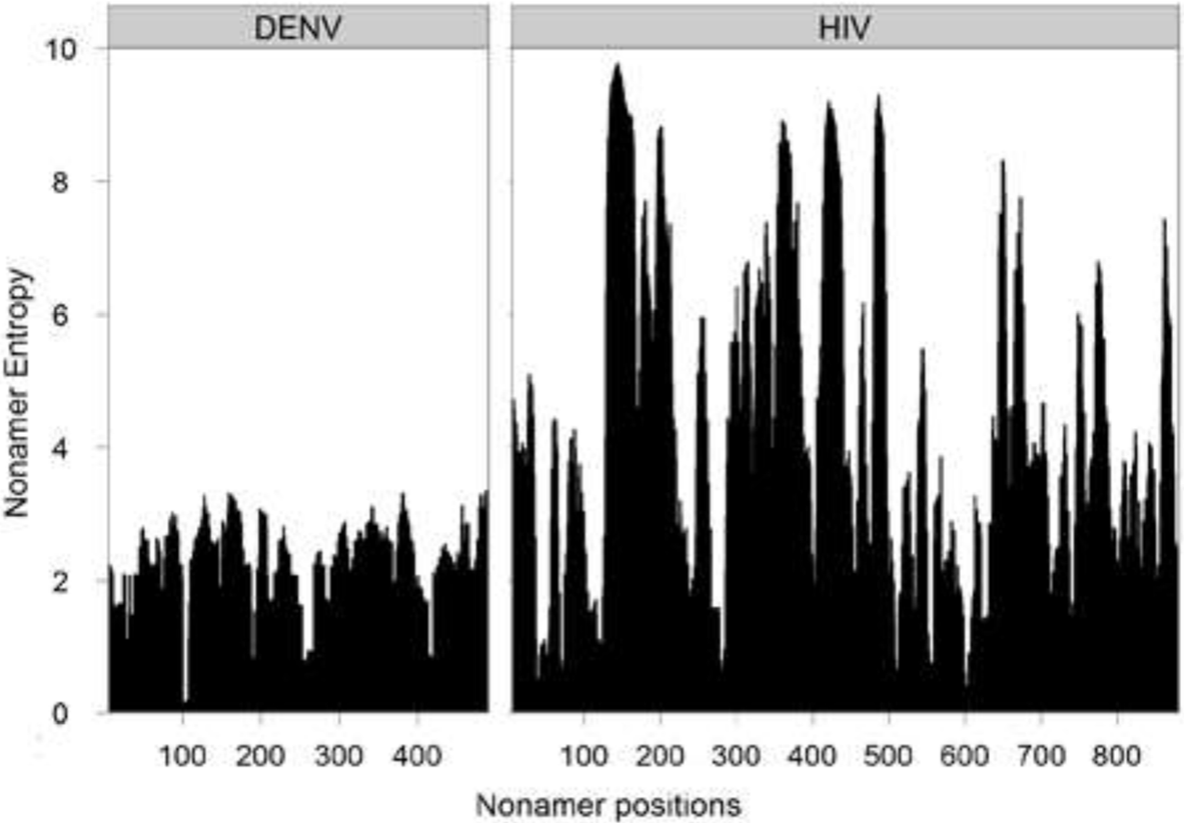
Entropy plot of nonamer peptide diversity for the envelope proteins of dengue virus (all four serotypes) and HIV-1 clade B. Large entropy values imply high variability, whereas closer to zero represent high conservation. HIV-1 envelope protein of clade B viruses is more diverse than the envelope protein of all reported dengue virus serotypes

1. Load the viral protein alignments to AVANA, either as aligned fasta sequences (.afa) or in tabbed alignment format (.taln). Aligned outputs from MUSCLE can be used directly as they are in “.afa” format, whereas those from PROMALS3D or Clustal X are in the typical “.aln” format and need to be converted to either aligned fasta (via Clustal X) or tabbed alignment (via BioEdit; See Tutorial 3).
2. Set AVANA to analyze nonamers within the alignment by selecting the “Preferences” option from the Tools menu, and defining “Sample Size” to “9” under the “Sequence Scan” tab. Ensure that under the “Diversity tab”, the default “Extrapolate entropy value to infinite sets” is checked to get entropy values corrected for size bias for each nonamer position, with “Number of random subalignment for extrapolation” set to “100”. Under the same tab, highly gapped positions are defined as those where the gaps are “at least 50% of the symbols”, and low-support positions as those with support of lower than “50% of the sequences”.
3. Output the corrected entropy values by clicking on the “View” menu and selecting “Variability” under the “Statistics” option. The output can be saved as a text file and opened in Excel or other text editor for tabulation and analyses.
4. Steps 1 to 3 are repeated for all the viral protein alignments, and entropy values of all overlapping nonamer sites for each of the viral protein alignments are plotted for panoramic view and analysis by use of the ggplot2 suite [45] of the R programming language and environment [46]. A sample plot for the entropy of the envelope protein of dengue virus (all four serotypes) and HIV-1 clade B is provided in Fig. 4.

### Variant analysis

The genotypic differences between strains of the primary and secondary viral infections, or between the strains of the vaccine and challenge infection, constitute a critical consideration for protective and, in some cases, pathologic immunity [23]. Because of intra- and inter-group sequence variability within a viral species, most T-cell epitope sequences may exhibit single or multiple amino acid differences within and between the groups. It is therefore important to also study viral diversity by taking into account the incidence distribution of sequences variant to the predominant (index) peptide at each of the aligned nonamer positions [2, 12, 15, 47]. Index nonamers are used as the reference sequences because they are peptides with the highest frequency (incidence) in the reported population of the virus species, and thus represent the peptides most likely of being recognized for presentation to T-cell, assuming that they are epitopes. Variants at a given position in an alignment are defined as all peptides with one or more amino acid differences to the index nonamer, and represent the population of altered peptide ligands that the immune system may eventually be exposed to after immunization with the index nonamer sequence of the position. Index nonamers with no or low variants incidence (~ <20%) are potentially useful in reducing the risk or avoiding the issue of altered peptide ligands. However, such index nonamer sites of low variants maybe dominated by a single major variant (~ >10% incidence), which should be avoided. Thus, besides studying the incidence distribution of all variants at a nonamer position, it is also important to study the distribution of a specific variant, the major variant.

**Fig. 5:**
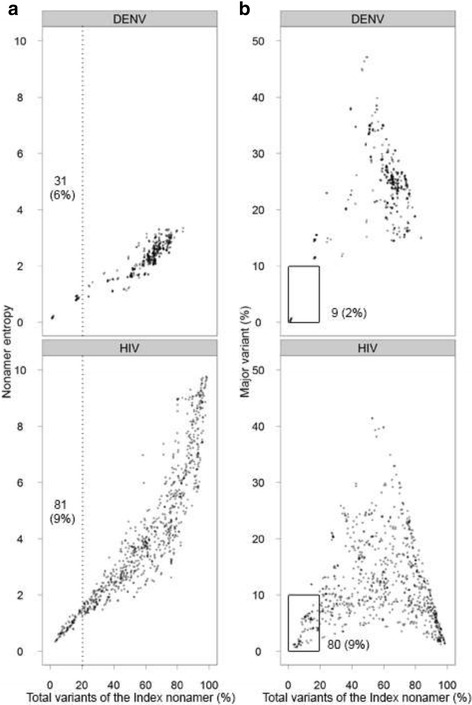
Variant analysis of the envelope proteins of dengue virus (all four serotypes) and HIV-1 clade B. **a** Density plots for the incidence of total variants of the index nonamer and the entropy of the nonamer sequences for the envelope proteins of dengue virus (all four serotypes) and HIV-1 clade B. The variants incidence is widely distributed for HIV-1 clade B, whereas for dengue it is mainly localized in the higher range, with very few positions (6%) under the 20% incidence region (dotted line). **b** Density plots for the incidence of all variants to the index nonamer and the major variant at each nonamer position for the envelope proteins of dengue virus (all four serotypes) and HIV-1 clade B. The boxed regions and the adjacent values indicate the fraction and number of total nonamer positions analyzed that are highly conserved, contain fewer than 20% variants of the index sequence and fewer than 10% incidence of the major variant. Despite the lower overall entropy of dengue virus (all four serotypes) compared to HIV-1 clade B, only 2% (9 nonamer sites) of the dengue virus envelope nonamers satisfied this criteria, while more favourable targets (9%; 80 nonamer sites) are present for the envelope of HIV-1 clade B

1. Repeat the AVANA analysis steps 1 and 2 described in section “Entropy analysis”.
2. Output the variant data by clicking on the “View” menu and selecting “Variants” under the “Statistics” option. The output can be saved as a text file and opened in Excel for tabulation and analysis. Note that in this output, the index nonamer peptide data is also included and should not be considered as part of the variant.
3. The variant output and the previous section’s entropy output for each aligned nonamer position document the following: the index nonamer sequence and its incidence, total variants incidence, number of distinct sequences that make up the variants, and the incidence of the major variant (an example is provided in Additional File 1). The distribution of total variants of the index nonamers is analysed against entropy (diversity) (sample output in Fig. 5a) and major variant (sample output in Fig. 5b) for each of the protein alignments. The ggplot2 suite is used to plot the distribution for the analysis.

### Analysis of distribution of natural variants

All nonamers of the virus species of interest should be studied for distribution in nature to assess for the extent of conservation of genetic sequences in the phylogeny of other virus species or organisms. The diversity analysis of several viruses suggests that it is prudent to select highly conserved sequences that are also virus species-specific, as epitopes common to other pathogens could be pathologic [21, 23, 48]. Virus species-specific nonamers are defined as those that do not share nine consecutive amino acid identities with sequences of other viruses and organisms. This is especially important with species that have the potential to or are known to co-circulate and/or co-infect. For example, dengue virus is known to co-circulate as different serotypes and with several of the other major human pathogen *Flaviviruses* [49, 50]. The definition of virus species-specific nonamers can be expanded to exclude those with one amino acid mismatch to human sequences in order to avoid possibility of molecular mimicry.

1. The distribution in nature of all the index nonamers and their variants for each of the protein alignments is analysed by scanning against the most comprehensive “nr” database at NCBI, which stores all reported protein sequences of all species to date. The compressed and preformatted version of this large nr database can be downloaded from BLAST+ Databases URL in the “Materials” section. After unzipping the downloaded nr files, the sequences can be extracted in fasta format from the preformatted nr files by use of the command below:

blastdbcmd -db <db file name> -entry all -outfmt %f -out <output file name>

- where “-db” is used to define the name of the nr database file and “-out” is to define the name of the output file where the fasta sequences will be saved. Note that the nr database file is downloaded in multiple files, and thus the above command needs to be repeated for each file.

2. An in-house Perl script is used to perform the scan of all unique nonamers of the virus species of interest against the nr database fasta file for exact matches, and another in-house Java program is used to extract the organism name for each of these matches and report a list of nonamer peptides that were found in other virus species and organisms, but excluding self-hits to the virus species of interest, synthetic constructs and artificial sequences. Researchers interested in the Perl script and the Java program can email their requests to us at vaccineinformatics@gmail.com.

An alternative to the Perl script approach is to perform BLAST search against the nr database, in particular when similar matches are desired in addition to the exact ones. First, BLAST searches, with parameters optimized for short sequence queries, are performed against all reported viruses as follows: search by Entrez query limited to “Viruses[ORGN] NOT <txid> NOT txid81077[ORGN]” where “<txid>” is the taxonomy ID of the species of interest, such as “txid11082[Organism:exp]” for West Nile virus, and artificial sequence hits are removed by the keyword “NOT txid81077[ORGN]”; “automatically adjust parameters for short sequences” option disabled, “low-complexity” filter disabled, maximum number of aligned sequences to be displayed set to “20,000”, expect threshold set to “2000”, or “20,000”, or “200,000” until a valid result was obtained, word size set to “2”, matrix set to “PAM30”, gap costs set to “Existence: 9, Extension: 1”, compositional adjustments set to “no adjustment”.

Next, similar BLAST searches are carried out against protein sequences of all organisms, where the parameters are the same as the previous search against all viruses, except that the search by Entrez query is limited to “All[FILTER] NOT Viruses[ORGN] NOT txid81077[ORGN]”. Only the distinct nonamers of the virus species of interest are used as queries, and may be concatenated together to reduce the number of BLAST search to one or few. However, this concatenation will result in artificial hits matching regions in between the nonamers, which are to be ignored during the processing of the results. The BLAST search can be performed at the NCBI database (see Tutorial 4 for notes); however, a limitation of this is that not all desired hits may be detected because a maximum of only 20,000 hits can be returned per search, where it is possible that false positive hits may push relevant ones beyond the 20,000 limit. This is why the single step web BLAST search with the Entrez query “All[FILTER] NOT <txid> NOT txid81077[ORGN]” may not be desired. Performing the search by use of the local BLAST client programs (BLAST+) and sending the request to NCBI “nr” database by use of the “-remote” option, which allows adding the command line option “-entrez_query All[FILTER] NOT Viruses[ORGN] NOT txid81077[ORGN]”, is subject to the same hit limit imposed by NCBI. This can be overcome by performing the search with nr database downloaded locally; see the BLAST+ manual regarding “-gilist” and the “blastdb_aliastool” to do this. The GI accession list can be generated by searching the above Entrez query keyword at the NCBI Entrez Protein Database.

**Fig. 6:**
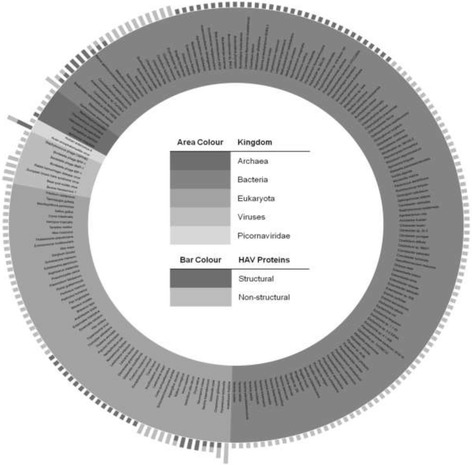
Distribution in nature of hepatitis A virus (HAV) protein nonamers. Nonamers of HAV are found across viruses, archaea, bacteria, and eukaryota kingdoms. Picornaviridae are viruses of the same family as HAV. HAV is a single stranded RNA virus that encodes for four structural proteins, namely 1A (VP4), 1B (VP2), 1C (VP3), and 1D (VP1), and seven non-structural proteins (2A, 2B, 2C, 3A, 3B, 3C and 3D). The length of the protein bars indicate the number of nonamers of these protein that are matched by the other species. Highly conserved nonamers that do not match any one of the species, in particular the co-circulating viruses are favourable for selection as potential vaccine targets in order to avoid potential altered ligand effects

A sample output of the natural distribution analysis for hepatitis A virus is illustrated in Fig. 6. Unlike *Flaviviruses*, such as dengue [12] and West Nile viruses [15], the conservation of hepatitis A virus sequences does not extend to many other viruses, making them hepatitis A specific. However, both families of these viruses share extensive conservation of peptides with eukaryotes and prokaryotes. Matches to prokaryotes maybe relevant if there is literature support for co-infection, while for matches to eukaryotes, only those that belong to humans are perhaps of interest.

4. In addition to exact nonamer matches to human sequences, nonamers of the virus species of interest with a single mismatch to human sequences at any position along the nonamer are analysed to assess the possibility of “molecular mimicry” by the virus. All human protein sequences are downloaded for the search from the NCBI Entrez Taxonomy Database (Taxonomy ID 9606), following the steps described in section “Data collection”. The “agrep” (approximate “grep”) Unix code is used to perform the one amino acid mismatch search with the human sequences. Functional annotations of the identified human proteins can be obtained by searching the UniProt database [51] for their Gene Ontology (GO) term distribution in the context of cellular component, molecular function and biological process. GO terms can be predicted by use of the BLAST2GO server [52] if annotation is not available at UniProt.

### Functional analysis

Highly conserved protein sequences generally represent important structural or functional domains [53], and their mutations would be detrimental to the survival and/or fitness of the virus. Therefore, it is important to assess possible correlation of function to conserved sequence regions for each protein of the virus species of interest. A correlation would suggest that function is important in maintaining the conservation of the sequences and these are likely to remain conserved in the future, making them favorable vaccine targets. This, however, may not be true if functionally important domains or motifs are also found to correspond to variable regions of the protein, as observed for HIV-1 clade B [54].

1. Reported and putative functional properties of the virus species of interest are identified by searching the literature and the UniProt database [51]. The Prosite [55], CCD [56] and Pfam [57] databases may also be accessed for additional functional information.
2. Identified functional information is compared against the entropy plot/data of each protein to assess whether there is correlation between function and conservation.

### Identification of virus species-specific, highly conserved sequences

Highly conserved virus species-specific sequences are identified as potential candidate vaccine targets if a good understanding of viral diversity can be attained through the analyses described in the previous sections. First, highly conserved nonamers are identified based on the analysis in sections “Entropy analysis” and “Variant analysis” before addressing the specificity issue based on the analysis in section “Analysis of distribution of natural variants”.

Depending on the conservation of virus species (sections “Entropy analysis” and “Variant analysis”), analysis can focus on identifying completely conserved nonamers (100% incidence in all analyzed sequences of the protein) for highly conserved viruses, such as West Nile virus. A total of 88 completely conserved sequences with entropy of zero were identified for West Nile virus [15]. For moderately diverse viruses, such as dengue virus, nonamers of more than 80% incidence (i.e. 20% variants) may be selected, depending on the number of the peptides within this range. Typically for these viruses, the major variants of each of the nonamers are present in less than 10% of the sequences analysed. However, this is likely to be higher for more diverse viruses, such as HIV-1 or Influenza A, and hence index nonamers with major variants incidence of more than 10% are ignored. For example, a total of 78 highly conserved sequences, with an index nonamer incidence of more than 80% and major variant of less than 10%, were identified for HIV-1 clade B proteome [2]. Pan-group conserved sequences are identified for viruses with multiple distinct groups (clades, serotypes or subtypes). This is done by first identifying conserved nonamers for each individual group at a defined threshold of index nonamer and major variants incidences, and then selecting resulting nonamers that are consensus between the groups. A total of 44 pan-group sequences were identified for dengue virus, which were present in ≥80% of all sequences of each dengue serotype group [12].

After identification of the conserved nonamers for the virus species of interest, such sequences not shared (exact match) with any other virus and organism are selected as potential candidate vaccine targets for experimental validation. Selected nonamers that are contiguous (overlapping by eight amino acids) are joined to form longer sequences. Sequences may be prioritized based on the analysis in section “Functional analysis”.

### Relevance to current circulating strains analysis

The relevance of the identified potential candidate vaccine targets for use against current circulating strains can be assessed by measuring the incidence of the candidate targets in the corresponding sequences of recent strains of the virus of interest, taking data over the past 10 years that represent broad geographical ranges. Candidate targets with nonamers that are highly represented in the current data are further prioritized for experimental validation. This analysis also provides insight into how the diversity of the current strains compares with that of all reported data.

1. Current circulating strains data of over the past 10 years with no geographical restriction is typically a subset of all processed collected data as described in section “Data processing” and can be easily extracted as country and year of isolation are noted (as required in step 2 of section “Data processing”). If a significant number of newer sequences have been deposited in public databases since the time of the data collection (section “Data collection”), these new sequences are collected and processed as described in sections “Data collection” and “Data processing” and merged with the relevant subset of the existing data.
2. The incidences of the nonamers of the candidate vaccine targets within the data of the recent circulating strains is obtained by performing analysis similar to that described in sections “entropy analysis” and “variant analysis”.

### Immune relevance analysis

Targets of immune response pre-selected by computational analysis minimize the number of experiments required for validation [58]. Web servers/tools based on a number of algorithms are available for reliable prediction of promiscuous or HLA-supertype restricted peptides. T-cell epitopes promiscuous to multiple HLA alleles of a supertype are advantageous for vaccine design because they are applicable to a large proportion of the population as individuals with different HLA alleles can respond to the same epitope [59]. Preference may be given to epitopes that are promiscuous to multiple supertypes as these sequences provide for a wider population coverage than those relevant to one supertype. Promiscuity to both HLA class I and II supertypes additionally provides immune relevance to both cytotoxic and helper T-cell responses. Regions of the viral sequence containing high concentration or clusters of promiscuous T-cell epitopes (immunological hotspots) [13, 60, 61], preferably of different supertypes, are also attractive because of the availability of multiple targets within the same sequence, which provide for broader human population coverage if relevant to multiple HLA supertypes, and cover a larger spectrum of the pathogen variants. Experimental measurements for validation of computational predictions are critical for accurate interpretation of results. Predicted promiscuous T-cell epitopes within the potential candidate vaccine targets can be validated by reports of experimentally confirmed T-cell epitopes from human, humanized mice, or laboratory mice studies, available in the literature or public databases. Data from human studies are ideal, while those from humanized mice are next in the order of reliability as correspondence with human data has been shown [62, 63], while laboratory mice data need to be taken with caution. Eventual experimental measurements may still be necessary to validate the immune relevance of the predicted promiscuous epitopes within the potential candidate vaccine targets, particularly for those with a little or no correspondence to published data. Validated potential candidate vaccine targets can then be subjected to subsequent clinical studies, such as validation in monkeys.

1. Putative HLA class I supertype restricted peptides within the potential candidate vaccine targets are identified by use of NetCTL [64], ARB [65], PEPVAC [66] and MULTIPRED2 [67–69], and for class II restricted by use of MULTIPRED2 and TEPITOPE [70]. ARB and TEPITOPE do not directly provide the HLA supertype binders, and the users would have to identify them by determining the peptides binding to multiple alleles of a supertype of interest with acceptable predicted affinity (see [12] for example). See [20, 71] for definition of HLA class I and II supertype alleles, respectively.
2. Search against both the available literature and epitope databases is performed for reported T-cell epitopes (both class I and II) of the virus species of interest from human, transgenic mice and lab mice studies that match at least nine amino acids of the predicted epitopes. Epitope databases include general ones, such as the Immune Epitope Database [72], SYFPEITHI [73], MHCBN [74], and specialized ones, such as HCV immunology database [75], HIV molecular immunology database [76], and influenza database, among others. PubMed or Google search should be carried out to identify specialized databases for the virus species of interest. Though the epitopes in the specialized database are likely to be found in the general databases, it is worth looking at the specialized databases as additional metadata may be provided or the data presentation maybe easier for parsing.
3. Experimental quantitative measurements of the potential candidate vaccine targets can be performed through functional assays, including HLA binding assays [77], immunization of HLA transgenic mice and ELISpot assay for peptide-specific T-cell activation [63] and of pathogen infected human subjects. The methods for these are widely available in the extant literature and thus are not described here.

## Discussion and conclusions

Traditional approaches to vaccine target identification in the past twenty years have required tedious and costly screening of peptides for their antigenicity and immunogenicity. Costs have declined but the traditional approach is still time consuming to cope with viral diversity. To improve the efficiency and productivity of the vaccine target discovery process, the approach of reverse vaccinology has improved the situation, leveraging on the in silico analysis of both the diversity of the virus and of the human immune system. In particular, with the deluge of sequence data emerging from powerful advances in DNA sequencing technologies in the recent past years, combined with high performance cloud and grid computing and broadband networks, data exchange and analysis have become more convenient than ever, frequently accessible to the ordinary bench researcher. While structural analysis of ternary complexes of T-cell receptor (TCR), peptide ligand and the major histocompatibility complex (MHC) have seen rapid progress; the process is still hampered by lack of data. Meanwhile, large-scale sequence analysis still offers an attractive and rapid route to viral vaccine target discovery. Using the methodology described in this article and the tools that are available, we make accessible a reverse vaccinology approach to other researchers in hope that such tools will enable more efficient vaccine discovery. In the near future, we anticipate that the availability of more human genomic sequences and a more comprehensive correlation of the sequence with the structural variations of the MHC and the TCR, will allow us to perform more comprehensive analyses for studying viral conserved sequences against a richer backdrop of both the MHC and the TCR.

## Additional file

Additional file 1: Table S1. Entropy and variant analysis data from a specific region of HIV-1 clade B envelope protein (Env). (PDF 87 kb)
