# Supplementary material for: Analysis of viral diversity for vaccine target discovery
Source: BMC Med Genomics. 2017 Dec 21;10(Suppl 4):78. doi: 10.1186/s12920-017-0301-2 (PMC5763473; doi:10.1186/s12920-017-0301-2)
Supplement: Additional file 1: Table S1. — Entropy and variant analysis data from a specific region of HIV-1 clade B envelope protein (Env). (PDF 87 kb) [file 12920_2017_301_MOESM1_ESM.pdf]

Additional File 1: Table S1. Entropy and variant analysis data from a specific region of HIV-1 clade B envelope protein (Env).

Position	Nonamers analysed *#		Index nonamer [c]		Variants of the index nonamers [d]		
	No.[a]	Nonamer entropy [b]	Sequence	Incidence [No. (%)]	Total Incidence [No. (%)]	Distinct[e] [No.]	Major variant incidence [f] [No. (%)]
397-405	3968	1.7	FNCGGEFFY	3107 (~78)	861 (~22)	106	281 (~7)
398-406	3960	1.7	NCGGEFFYC	3118 (~79)	842 (~21)	107	282 (~7)
399-407	3902	2.0	CGGEFFYCN	2875 (~74)	1027 (~26)	108	273 (~7)
400-408	3902	2.8	GGEFFYCNT	1849 (~47)	2053 (~53)	131	1028 (~26)
401-409	3901	3.0	GEFFYCNTT	1831 (~47)	2070 (~53)	142	794 (~20)
402-410	3898	4.7	EFFYCNTTQ	800 (~21)	3098 (~79)	236	453 (~12)
403-411	3898	4.7	FFYCNTTQL	805 (~21)	3093 (~79)	225	456 (~12)
404-412	3898	4.7	FYCNTTQLF	810 (~21)	3088 (~79)	224	458 (~12)
405-413	3897	4.9	YCNTTQLFN	796 (~20)	3101 (~80)	260	449 (~12)
406-414	3726	5.1	CNTTQLFNS	717 (~19)	3009 (~81)	265	429 (~12)
407-415	3737	5.5	NTTQLFNST	607 (~16)	3130 (~84)	320	416 (~11)
408-416	3732	5.2	TTQLFNSTW	665 (~18)	3067 (~82)	298	424 (~11)
409-417	3727	6.5	TQLFNSTWN	539 (~14)	3188 (~86)	426	306 (~8)
410-418	3725	7.7	PLFNSTWGS	262 (~7)	3463 (~93)	603	160 (~4)
411-419	3701	7.3	LFNSTWGSN	261 (~7)	3440 (~93)	518	246 (~7)
412-420	3635	8.3	FNSTWGSND	257 (~7)	3378 (~93)	694	88 (~2)
413-421	3633	8.6	NSTWGSNDS	189 (~5)	3444 (~95)	800	67 (~2)
414-422	3632	8.9	STWGSNDSR	161 (~4)	3471 (~96)	875	67 (~2)
415-423	3630	9.0	TWGSNDSRP	159 (~4)	3471 (~96)	942	63 (~2)
416-424	3628	9.1	WGSNDSRPE	157 (~4)	3471 (~96)	984	63 (~2)
417-425	3613	9.2	GSNDSRPEN	155 (~4)	3458 (~96)	1019	63 (~2)
418-426	3610	9.1	SNDSRPENN	192 (~5)	3418 (~95)	993	63 (~2)
419-427	3595	9.1	NDSRPENNT	212 (~6)	3383 (~94)	966	63 (~2)
420-428	3567	9.1	DSRPENNTG	212 (~6)	3355 (~94)	983	63 (~2)
421-429	3346	9.1	SRPENNTGG	209 (~6)	3137 (~94)	924	63 (~2)
422-430	3055	9.1	RPENNTGGN	254 (~8)	2801 (~92)	840	63 (~2)
423-431	2411	8.9	PENNTGGNE	308 (~13)	2103 (~87)	629	60 (~2)
424-432	1945	9.0	ENNTGGNET	289 (~15)	1656 (~85)	493	60 (~3)
425-433	1232	8.9	SENTTGNGT	60 (~5)	1172 (~95)	363	38 (~3)
426-434	825	8.8	TEVKNNTEG	38 (~5)	787 (~95)	256	28 (~3)

[a] The total number of HIV-1 clade B protein sequences obtained at the respective nonamer positions of the protein sequence alignment. The number of sequences for each nonamer position varies due to the inclusion of both partial and full-length sequences.
[b] Shannon's nonamer entropy.
[c] The index nonamer is the peptide with the highest incidence at a given nonamer position in the protein alignment.
[d] Variants of the index nonamers are all sequences that differ by one or more amino acids from the index nonamer at the corresponding position in the protein alignment.
[e] The number of distinct variants at the indicated nonamer position.
[f] The major variant is the most common (highest incidence) variant nonamer of all analyzed nonamers at the indicated nonamer position of the protein alignment.
[*] An example interpretation of the table: The index nonamer FNCGGEFFY was present in 3107 sequences (~ 78%) of all 3968 sequences analyzed at nonamer position 397-405 in the Env protein alignment. The remaining 861 sequences (~ 22%) at that position were variants of the index nonamer and comprised 106 distinct peptides, one of which is the major variant and is present in about 7% (281) of all the 3968 analysed sequences. The remaining 105 distinct variants at the position were represented by 580 additional variant sequences.
[#] This example shows a region of lower entropy, positions 397-405 : 398-406 with entropy below 2.0, which is connected to positions 412-420 : 426-434, a region of high diversity (entropy above 8.0), by a transitional region of intermediate entropy.
